# FingerScanner: Embedding a Fingerprint Scanner in a Raspberry Pi

**DOI:** 10.3390/s16020220

**Published:** 2016-02-06

**Authors:** Jordi Sapes, Francesc Solsona

**Affiliations:** Department of Computer Science and INSPIRES, University of Lleida, Jaume II 69, Lleida 25001, Spain; jordisapes@gmail.com

**Keywords:** embedding, Raspberry Pi, fingerprint, website

## Abstract

Nowadays, researchers are paying increasing attention to embedding systems. Cost reduction has lead to an increase in the number of platforms supporting the operating system Linux, jointly with the *Raspberry Pi* motherboard. Thus, embedding devices on *Raspberry*-Linux systems is a goal in order to make competitive commercial products. This paper presents a low-cost fingerprint recognition system embedded into a *Raspberry Pi* with Linux.

## 1. Introduction

This project consists of the development of a low-cost and competitive security environment of fingerprint recognition based on a GT (511C1R) device, and embedded into a *Raspberry Pi B+* (from now on, it is denoted as *Raspberry*) with *Raspbian* Linux.

This work presents a preliminary study about the viability of integrating a fingerprint device and a *Raspberry* with Linux into the same framework and, at the same time, providing a user interface by means of a web server. This first prototype, called *FingerScanner*, is a security system that provides the users a means to be validated by using a fingerprint scanner. *FingerScanner* can then be used to build much more complicated systems on top of it. However, we are interested in focusing our attention on designing an efficient prototype with a competitive performance.

This manuscript can be the basis for other possible projects that encourage *Raspberry* and similar boards developers to create interesting projects about accessibility, security, etc, combined with low-cost fingerprint scanners. A sample project could be a safe deposit box with a *FingerScanner*. Nowadays some enterprises in the sector of cash handling that use finger-print sensors (*i.e.*, Sallén, http://www.sallen.es) complain about the fingerprint tools (to develop an application) and sensor cost. So, our project can become the basis of the low-cost systems based on fingerprint sensors.

The remainder of this manuscript is organized as follows. Section 2 details the related work and motivation as well as the contributions of this paper. Section 3 introduces the main features of the devices used in this project. Section 4 presents the design of the proposed *FingerScanner* application. The experimentation described in Section 5 evaluates the performance and efficiency of our proposed solutions. Finally, Section 6 outlines the main conclusions and future work.

## 2. Related Work

Nowadays, there is a growth in *Raspberry* projects. There are many interesting projects similar to ours. Website [1] is full of *Raspberry* projects in many different areas. We only highlight one of them, *Node-pi-alarm* [2], where sampling data obtained from cameras and sensors are displayed on a website. The main similarity with our project is that the web server was made in *Node.js* inside the embedded system.

The authors in [3] present a security home system based on the *Internet of things*. However, security issues concerning the management of a huge number of connected devices cost effectively has emerged in this research field. This article presents a system to connect a door to Internet, so that the access control system can be controlled from anywhere in the world. It provides such facilities as visitor recording, notification and chat with the administrator and remote door lock/unlocking. Communication is based on Twitter and email. This system uses a *Raspberry* and manages various sensors via Internet. The idea presented can be extended from our *FingerScanner* prototype.

In [4], a real-time monitoring system for a fire alarm was proposed that detects and takes pictures in the presence of smoke, called *Firefighter*. The embedded systems used to develop this fire alarm system were *Raspberry* and *Arduino Uno*. The key feature of the system is the ability to display an image of the room state on a web page when a fire is detected through the presence of smoke. The system needs user confirmation to report the event to the *Firefighter* server through an SMS message. The advantage of using this system is that it reduces the possibility of false alerts reported to the server. The drawback is the human interaction required to take decisions and the high power consumption. In addition, the real-time need forces the use of a non-free SMS messaging system. As our project, it is based on a *Raspberry* and uses a web page as the visual information system. However, the use of SMS messages is a good solution to implementing real-time communication systems.

In [5], a design and implementation method for RESTful WSN Gateways was proposed using *Node.js*, as in our project. The gateway is designed as an embedded Linux device, which can handle multiple accesses with both sensors and servers. As in our project, it used the *Raspberry* and a web page as the visual information system. The real-time requires the use of an SMS messaging system.

In [6], a sensor secure node server to communicate over Bluetooth using RC4 encryption algorithm between a mobile phone and its monitoring equipment was proposed. An analogous idea should be incorporated into our system to provide more secure communications.

In [7], pressing an alarm switch enables a PIR sensor and a USB camera is turned on for 10 s and records the face of the person at the door. The captured video is then transmitted to the administrator through a 3G dongle with a unique IP address.

Other papers also deal with the design and implementation of smart surveillance monitoring system using *Raspberry* and some sort of sensors [8] and alert with SMSs messages [9].

In [10], the *Raspberry* operates and controls motion detectors and video cameras for remote sensing and surveillance. For instance, when motion is detected, the cameras automatically initiate recording and the *Raspberry* device alerts the homeowner of the possible intrusion through a web page. In general, globally accessible automation of electronic appliances in a user friendly way can be made possible with the use of a *Raspberry* micro-controller board, an Internet connection and relay switches [11].

The authors in [12] developed a methodology with low time computational complexity for detecting fingerprints, where a support vector machine (SVM) learning method was proposed. High computing performance required in detection, where instant system responses are required was shown in a project where real-time face detection on *Raspberry*’s graphic processor was presented in [13].

The authors in [14] explain how to create an intelligent, high-efficiency street lighting system based on a *Raspberry*. In this case, the authors linked the *Raspberry* to two *ZigBee* sensors to switch the streetlights on and off.

A new inexpensive vineyard protection against hailstorm was presented in [15]. Each row has an “umbrella” which protects the product without hindering the mechanical activities. It had an electronic card and a *ZigBee* mesh telecommunication network to transmit data to a *Raspberry* Pi that manages the protection.

As another example of low-cost solutions, the authors in [16] presented a multi-parameter acquisition system for volcanic monitoring also based on the *Raspberry*. The acquisition system was developed using a System on a Chip (SoC) Broadcom BCM2835 Linux operating system (based on DebianTM) that allows for the construction of a complete monitoring system offering multiple possibilities for storage, data-processing, configuration, and real-time monitoring of volcanic activity.

Almost all the cited projects (like our own) rely on the communication performance provided by the *Raspberry*. In [17], *Raspberry* was shown as a cheap, flexible, fully customizable and programmable small computer embedded Linux board. In [18], measurements showed that processing rates of up to 230 Mbps and the energy consumption per bit with the *Raspberry* can be as small as 3 nJ/bit. Then, *Raspberry* was shown to be an inexpensive, viable and flexible platform to deploy networking projects on.

Although all of them were well-structured proposals, none of them analyzed the efficiency of the system in depth. We measured the CPU and Memory of the embedding system used (*i.e.*, *Raspberry*) and different alternatives to implement the application presented (*FingerScanner*) were analyzed. In addition, an in-depth analysis of the browser (where controlling messages are displayed) was performed. In addition, special attention was paid to finding the most efficient devices, in other words, those with the best quality/price relation. This is an important requirement in manufacturing industry because the final products must be as competitive as possible.

## 3. Preliminary Concepts

**Figure 1:**
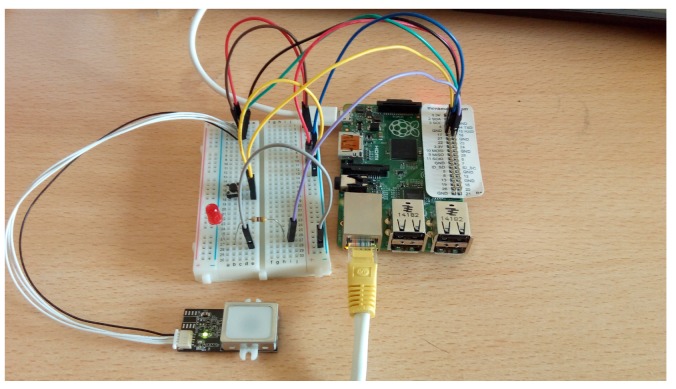
This image shows the physical connections between the *Raspberry* and the peripheral devices. The LED emulates the magnetic lock. An Ethernet connection is also needed because the *Raspberry* runs a *Node.js* web server.

**Figure 2:**
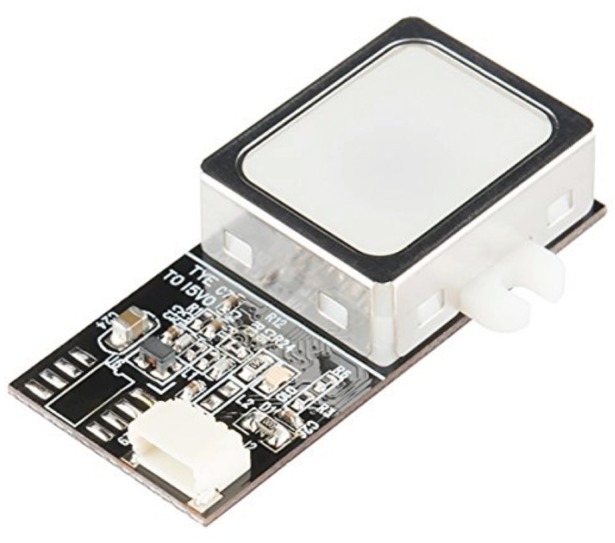
Fingerprint Scanner GT(511C1R).

The main components of our framework are (see Figure 1):

- *Raspberry Pi B+*. Due to its characteristics, this was the motherboard selected from among others (BeagleBone Black, HummingBoard, MinnowBoard Max, Pengwyn, Hachiko Board, *etc.*).
- The *Fingerprint Scanner GT(511C1R)* (Figure 2). This device uses 4-pin connector to communicate with the *Raspberry*.
- *JST SH Jumper 4 Wire Assembly*. This is the fingerprint Scanner connector. It has 4 pins to connect Voltage (3, 3–6 V), ground, TX and RX of different devices (in our case, the scanner and the *Raspberry*).
- The *Douself Sola* magnetic lock. When receiving a voltage of 12 V, it joins the two formed pieces. When voltage goes down, the two pieces separate.
- *Simple red LED*. It emulates the *magnetic lock*. The magnetic system is activated (deactivated) when the light is on (off).
- *Simple switch component*. It activates the scanner when pressed.
- The main programming language was *Node.js*.

The main features are explained below.

### 3.1. Raspberry

Nowadays, *Raspberry* is one of the most popular embedding systems with Linux support. The *Raspberry* community has grown continuously since its creation. It is a low-cost embedded system so some of the supported operating system are based on Debian, an open-source Linux distribution. It provides a very fluent developing software that you can downloaded from the *Raspberry* website (https://www.raspberrypi.org).

Raspberry Pi B+ (the model selected in this project) supports the following operating systems: *Raspbian*, *Snappy Ubuntu Core*, *Openelec*, *Raspbmc*, *Pidora* and *Risc*. We chose *Raspbian* because it is one of the most stable and well-documented operating systems. *Raspbian* is a Linux Distribution based on Debian Wheezy. We used a pre-compiled version for the *Raspberry*, which includes the basic developing tools, such as the Advanced Package Tool (APT).

### 3.2. Fingerprint Scanner Device GT(511C1R)

We chose the Fingerprint Scanner Device GT (511C1R) because it is very cheap and provides a well-documented manual, a Linux-compatible module as well as a good price/quality relation. More precisely, it provides a high-speed, high-accuracy fingerprint identification using the SmackFinger 3.0 Algorithm. It provides a 32-bit CPU at 72MHz (ARM Cortex M3) and a database which can store up to 20 different fingerprints. It is able to recognize a fingerprint in whatever 360${}^{{}^{\circ}}$ position. Downloads and uploads of fingerprint scans can be done by using the RS-232 serial interface. It provides a UART (Universal Asynchronous Receiver/Transmitter) connector (Default 9600 baud). Communication based on an UART communication protocol can operate through a serial RS-232 cable, the one chosen for this project. Power is supplied through a JST-SH connector.

The *Fingerprint* communicating protocol is based on packet handshaking. There are three kinds of packet:

1. Command packets: Used to order the device to carry out operations (*i.e.*, check for finger-button pressing).
2. Response packets: They indicate operation success/failure. The opcode of the command field can be ACK(0x30) and NACK(0x31), indicating operation success and failure respectively. In case of failure, the ERROR code is also provided.
3. Data packets: The data field does not have a static length because this packet is used to send extra information, fingerprint images, *etc.*

As a summary of functions, the *Fingerprint* scanner is small, cheap and gives accurate and rapid identification of fingerprints with an on-board optical sensor, stores 20 fingerprints in its database, and allows a single entry or the entire database to be downloaded and uploaded, among other features.

### 3.3. Software

We used *Node.js* as the main programming language. *Node.js* uses an event-driven, non-blocking I/O paradigm which makes it lightweight and efficient. It is perfect for data-intensive real-time applications and underpowered devices.

There are many modules Module: program component, linked dynamically to a program with the linker. Programs are composed of one or more independently developed modules that are not combined until the program is linked. A single module may contain one or several functions. Available for *Node.js*, such as the *Buffer* module, which allows communicating with the devices in the assembler. Among other reasons, *Node.js* was selected because it provides facilities to develop a driver for the *FingerScanner*. A module is made up in turn by a set of specialized functions. The modules can be written in *JavaScript*, and can be installed with the help of the npm utility.

To implement the asynchronous communication between the server (*Node.js*) and the *scanner* device, the *Buffer* module was used. Another possibility was to use *addons*. An *addon* is a dynamic shared object link written in C/C++, which integrates the V8 engine (a Google open-source library). With the help of V8 engine, C++ objects can be transformed into JavaScript code, which in turn are compatible with the *Node.js* application.

We discarded the *addons* option due to its difficulty and slowness. In this project we used the *Buffer* jointly with the *Promise* module, both integrated into a more generic module, *gt5113*, which also includes the *Serialport* module.

Finally, controlling the *FingerPrint* scanner with *Node.js* enables its features to be extended easily. For these reasons, we developed the *FingerScanner* application with *Node.js*.

## 4. FingerScanner

*FingerScanner* is a security system that provides a means to validate registered users by using a fingerprint scanner.

**Figure 3:**
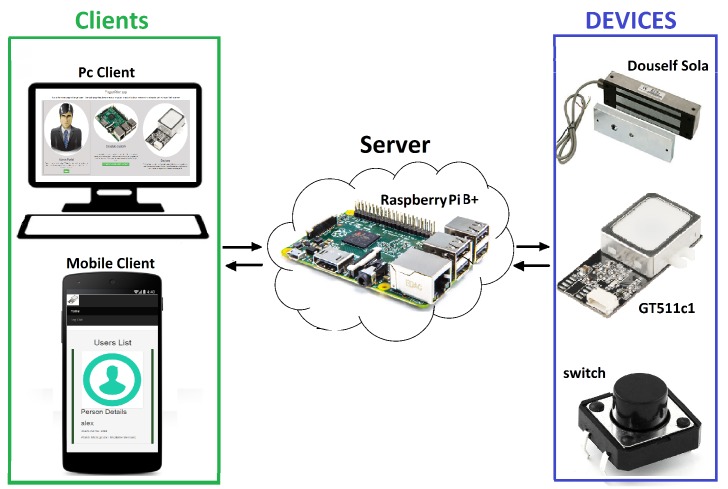
*FingerScanner* architecture.

The *FingerScanner* application follows a server-client paradigm. The client (which can be executed in a computer, tablets, smartphone and even in a *Raspberry* browser) makes requests to the server and this replies to the client. An administrator controls the system by using a web application that acts as the client. The server (running in the *Raspberry*) and implemented with *Node.js*), is responsible for managing the overall system, the *Raspberry* and their components and the devices attached to the *Raspberry*. Figure 3 shows the *FingerScanner* architecture. The *FingerScanner* components, client, server and devices (magnetic lock, fingerprint scanner GT511C1R and switch) can be seen, as well as their communicating links.

The administrator can register, delete and update users. The administrator, by means of a web page, sends user data to the server, which stores and interacts with a fingerprint scanner to perform the action requested from the client.

If a registered user wants to be validated, they must press a switch to activate the fingerprint scanner. If validated, the *FingerScanner* server unlocks the magnetic lock. Otherwise, the magnetic lock remains locked (action by default). If, for example, the magnetic lock is connected to the security system of a safe, this can control access to it.

In order to reduce the server work, the *Angular.js* framework was used to implement the client. *Angular.js* allows DOM (Document Object Model) objects to be manipulated, avoiding requests to the server. With the help of *Angular.js*, the client was implemented as a dynamic web. The main difference between a dynamic and non-dynamic web is the way the required client services are sent back from the server. When a user clicks on a link in a non-dynamic web, the server replies with an HTML page and its associated *JavaScript* code. Instead, in a Dynamic web, the server replies contain only data and the client is responsible for rendering the page. This greatly reduces the server work.

The server (implemented with *Node.js*) is responsible for receiving and dealing with signals from different devices (clients, switch and fingerprint scanner) connected to the *Raspberry*. To respond to inputs from devices or clients, *Node.js* implements *event listeners Event Listener*. Program (or function program) activated asynchronously by an event, that execute on the asynchronous arrival of their associated events.

The server stores registered users in a little relational **SQLite** database. SQLite is a small database used on systems with limited memory. The size of the database occupies up to 6 KB (when it stores 20 fingerprints). It uses a packet-exchanging serial communication with the *Fingerprint* scanner. It also controls the GPIO (General Purpose Input/Output) pins, through which the signals to/from the switch and the magnetic lock are sent/received.

The server activates (closes) the *Magnetic lock* when pin 12 voltage is 5 V (Logical 1). With 0 V (Logical 0), it is deactivated (opened). When the Identify process in the *scanner* ends successfully, the server sends the event “Logical 0” to the “Magnetic lock” pin to open it for a period (3 s). The module *rpi-gpio* is used to control the *Raspberry* pins. This module catches the voltage changes in the input pin (switch pin) and sets “Logical 0” or “Logical 1” into the output (magnetic lock) ones. Thus, the *switch* and *Magnetic lock* devices are controlled by the *rpi-gpio* module that catches and sends voltage changes to the pins (7 for switch pin and 12 for Magnetic lock pin)

The *FingerPrint* scanner is controlled differently. It is connected to the serial port *TTYAMA0* (pins TXD and RXD). The device receives input packets from the server (located in the *Raspberry*) and then returns response packets to the server using the serial port. This device has a small database that can store up to 20 fingerprints. Every scanned fingerprint has an associated ID (Identifier), which holds additional information about the fingerprint, saved in the server database (the SQLite one).

To deal with communication with devices and the client, it was necessary to install the following modules: *Express*, *gt511c3*, *Promise*, *SQLite*, *SerialPort* and *rpi-gpio*.

### 4.1. Communication Client-Server

In this section the communication protocol between the client and the server is presented. The client sends requests to the server by means of sentences formed by a *HTTP method* and an URI. An *HTTP method* indicates what the server has to do with one of the *URI*s (Uniform Resource Identifier) listed in Table 1.

**Table 1:** HTTP Methods.

Method	URI	Description
**GET**	domain/fingerprints	Obtain all information of all fingerprints
	domain/fingerprints/id	Obtain all information of one fingerprint
	domain/fingerprints/identify	Obtain information of the finger on the sensor
**POST**	domain/fingerprints	Save new fingerprint
**PUT**	domain/fingerprints/id	Update one fingerprint
**DELETE**	domain/fingerprints	Erase all information of all fingerprints
	domain/fingerprints/id	Erase all information of one fingerprint

There are different *Angular.js* controllers in the client. The *Angular.js* controllers are objects that allow the client logic to be developed as well as giving full control of the data. These controllers (made in *JavaScript*) perform specific functions inside the web page. Every controller has associated a *JavaScript* function activated asynchronously when users perform an action in the web. Some controller utilities implemented in our project are requests to the server for resources, show/hide elements in the DOM (Document Object Model), verify user data, *etc.* In other words, the interface sends requests to the server and the responses are managed by *Angular.js* controllers. As an example, *usersController* administrates the incoming server petitions requesting the registered users, or modifying or deleting a registered user. Depending on the server response (*i.e.*, success or failure), the controller alerts the user with a message on the web browser that everything went well or the occurred error. The other controllers implemented are *enrollController*, which controls the *Enroll* process (explained in Section 4.2.1) and *loginController* that only allows admin access.

The *Express* module obtains the client request. *Express* provides facilities to parse the information (body request, parameters, query in the URL, ID, *etc.*) of the client request. When the server receives a new request, *Express* launches a thread with a URI as argument. The server will execute the action associated to such a URI. Table 1 shows the action server associated with each URI. For example, with the GET method and URI *domain/fingerprints*, the client is asking for registered fingerprints. The server will send back all the fingerprints entries to the client. In this example, the client is only requesting data, but sometimes the client must send extra information. Additional information may be requested by the server in the POST and PUT methods.

According to the elements involved, the client requests (and more specifically their URIs) can be classified into three groups:

1. URIs where the server communicates with the local SQLite database (GET domain/fingerprints/, GET domain/fingerprints/id).
2. URIs where the server communicates with the FingerPrint scanner (DELETE domain/fingerprints/, DELETE domain/fingerprints/id).
3. URIs requiring human interaction.

It should be mentioned that two routes were not implemented because they are meaningless. These were *POST domain/fingerprints/id* and *PUT domain/fingerprints* (see Table 1).

### 4.2. Communication Server-Devices

In this section, the communication between the *Raspberry* and the different devices (switch, magnetic lock and fingerprint scanner GT511C1R) is presented.

The switch and magnetic lock are connected to the GPIO pins. The server knows which pins are connected by using the *rpi-gpio* module. This module can set the direction of the two GPIO pins (the *input* and *output* ones) and capture the signals produced in these pins. Thus, using the *rpi-gpio* module, the switch is assigned to the *input* pin and the magnetic lock to the *output* one.

An *event listener* is assigned to the switch pin. When caused by human interaction with the switch, the voltage in the GPIO pin changes. Then, the event listener is called. This voltage change means that a human user wants to be identified. The server captures the event in the GPIO pin. This informs the scanner that a new identification process must begin.

The *output* pin, assigned to the magnetic lock changes its voltage to activate or deactivate the magnetic lock. This change informs the user that the identification process has been successful.

The fingerprint scanner operation is more difficult than the other two devices. It is governed by a communicating protocol (based on the packet-exchanging handshake) between the server and the Fingerprint scanner device.

The command set used in the communication protocol (commands, errors, arguments, *etc.*) of the Fingerprint scanner is explained in depth in *(https://goo.gl/RwWdg4)*. The messages are made up of one or two packages (depending on whether data is/is not needed):

- **Command Packet (10 Bytes)**: Packet sent from the computer to the scanner. It contains: scanner Id, instruction, arguments and checksum.
- **Response Packet (10 Bytes)**: Packet sent from the scanner to the computer. It contains: response, scanner ID, checksum, and ACK or NACK indicating operation success or failure respectively (when NACK, it also contains the error code).
- **Data Packet**: Additional package of data to command or response packets. Its size depends on the data transmitted and it is used in the following operations: Open(opcional), MakeTemplate, GetImage, GetRawImage, GetTemplate, SetTemplate.

Fortunately, a module named *gt511c3* was recently released. The *gt511c3* uses two other important modules, *Promise* and *Serialport*. The *gt511c3* module implements the overall commands (the command packet and each corresponding response packet), which are in turn grouped into functions. All these functions return a *Promise* (an object provided by the *Promise* module), which contains the error code.

The *Promise* module is also used to implement the sequential code. There can be many threads executed in parallel running *Node.js* functions, as long as they are activated asynchronously. The *Promise* module can organize parts of the application to be executed sequentially in order to have more control over the asynchronous execution.

The *Serialport* module provides functionality to configure serial communication (baudrate, databits, stopbits, *etc*), and initiate and finish the serial communication. In our case, only the baudrate (115,200 or 9600 baud) was changed.

The main process steps of the server-scanner communication are:

1. *Enroll* (Record) a new fingerprint.
2. *Delete* one or more fingerprints from the DB scanner.
3. *Identify* that an imprint is already properly stored in the BD scanner.
4. *Update* a fingerprint is composed first by a *Delete* and then by a *Enroll* process.

The communication handshake between the scanner and the *Raspberry* in each of these three operations is explained next.

#### 4.2.1. Enroll

**Figure 4:**
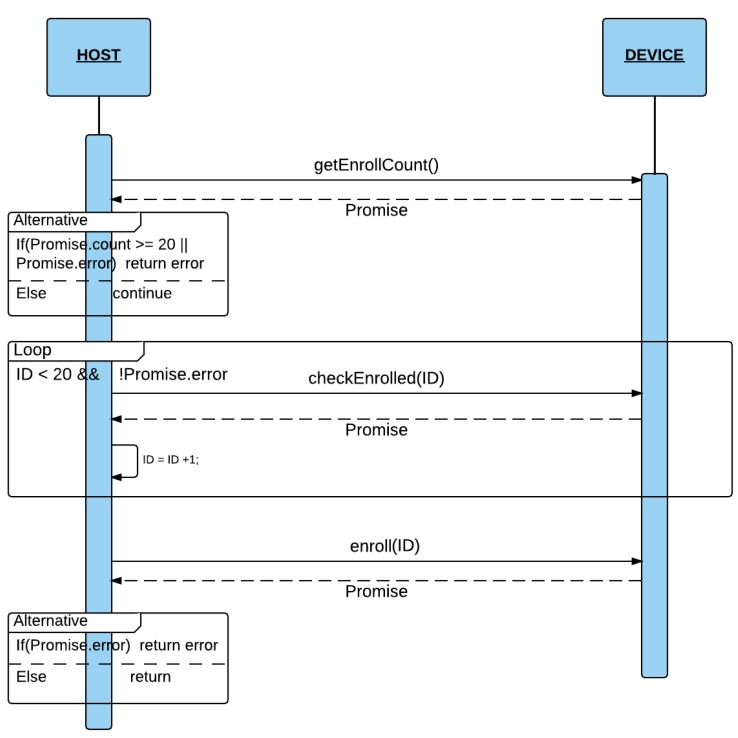
Enroll Process.

The Enroll process (see Figure 4) consist of obtaining a fingerprint scan 3 times with the LED on, and saving the image formed by merging these three scans in one of the 20 entries in the database device. Before calling the *Enroll* module function, it is necessary to obtain the occupied entries in the database by means of the *getEnrollCount* function. If the returned value is lower than 20, then the function *ckeckEnrolled* is called changing ID parameter, until the function does not return any error. The error means that the ID passed as a parameter is already stored in the scanner database. It returns the first empty entry (from 0…19) from the scanner database. Finally, the *Enroll* function with the ID entry number obtained as a parameter is called.

First of all, the *Enroll* function of *gt511c3* switches on the LED and wait for a finger scan up to a predetermined timeout (10 s). After receiving the scan, the scanner device saves it in Memory. Then, it switches off the Led. Thus, when the LED is on, it means that the device is performing a scan. This process is repeated 3 times and then the scanner merges the 3 scans in only one image.

The *Enroll* function is implemented by the following functions in the *gt511c3* module:

- *ledONOFF*: sends a signal to turn the LED on/off.
- *EnrollStart*: Allocates Memory space for *Enroll1*, *Enroll2* and *Enroll3*, and checks if the ID (passed as argument) is already saved in the DB. If so, the Enroll process will finish returning the fingerprint ID.
- *Enroll1*, *Enroll2*: Obtains the scan of what is on the scanner and saves it in Memory. The LED must be on.
- *Enroll3*: Obtains the scan of what is on the scanner and saves it in Memory. The LED must be on. Then, it merges the three scans obtained with *Enroll1*, *Enroll2* and *enroll3*.

#### 4.2.2. Identify

**Figure 5:**
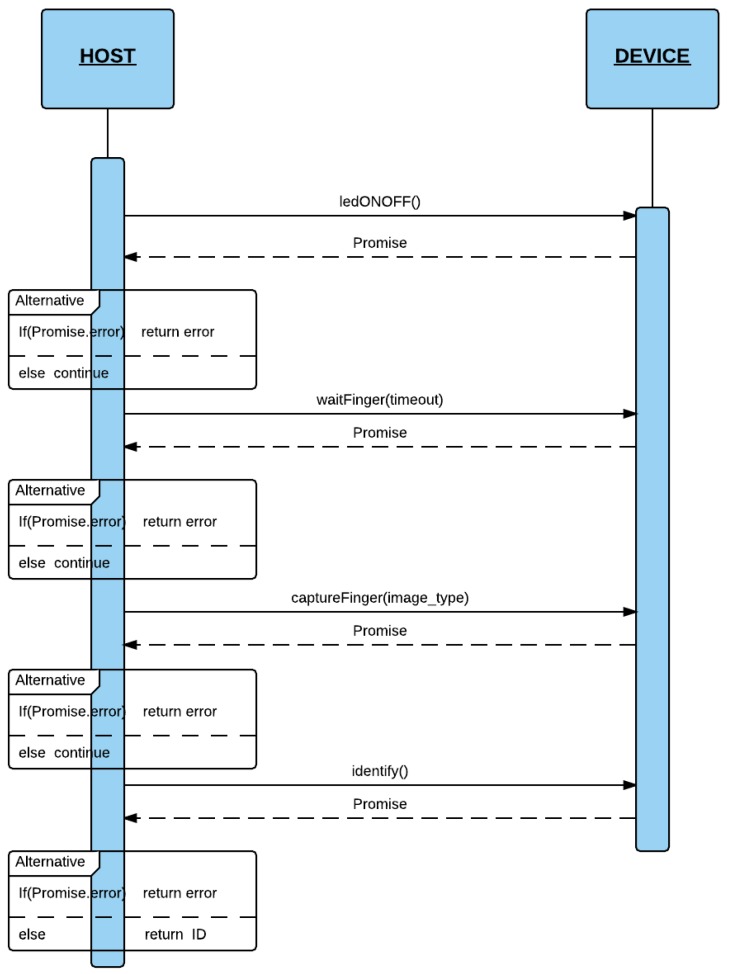
Identify Process.

In this section, the identification process (dentify process) is presented. Figure 5 shows the process steps for identifying a fingerprint.

First, the scanner switches on the LED (by using the *ledONOF* function). Next, the server waits for a finger to be placed on the scanner (*waitFinger* function). When the finger is on the scanner, this informs the server accordingly. If successful (*waiFinger and ledONOFF*), the server sends a command packet with the *captureFinger* command using the *captureFinger* function. On receiving this, if a NACK is received inside a *Promise*, the server obtains the error code and decodes the error. In other cases (all were gone successful), the server runs the *Identify* function that sends the *Identify* command. Finally, the scanner returns the ID associated with the finger. If the finger is not in the database, it returns an error meaning Identification failure. Then, the server makes a query to its local SQLite database to obtain more information if the user is already registered. When the process has been completed successfully, the server switches off the LED.

#### 4.2.3. Delete

Basically, the Delete process consist of sending a *Delete* command packet (*deleteID or deleteAll* packets) from the server to be removed from the Fingerprint scanner database. Upon receiving an encapsulated ACK from the scanner in a response packet, the *Raspberry* knows that the fingerprint has been removed successfully. The *deleteID* (passing the ID as argument) and *deleteAll* functions of the *gt511c3* module delete one or all the ID fingerprint, respectively. These functions can produce the following errors in the *Promise* object: NACK_INVALID_POS and NACK_EMPTY_DATABASE. When the process finishes successfully, the SQLite database of the server changes the same ID fingerprint entries accordingly.

## 5. Results

We performed various experiments to test the computational performance of *FingerScanner* to validate its viability. First, the performance of the communication system was measured to obtain the response times. Then, the behavior of the different components of the system (client, server and scanner) was analyzed in depth.

### 5.1. Communication Client-Server-Device

Table 2 shows the response time of the server performing the URIs.

**Table 2:** Response time server.

Route	Time (in Seconds)
GET domain/fingerprint	0.113
GET domain/fingerprint	0.104
GET domain/fingerprint/identify	4.5
DELETE domain/fingerprint	2.413
DELETE domain/fingerprint/id	2.587
POST domain/fingerprint	7.1
PUT domain/fingerprint/identify	8.457

We separated the results into three categories. URIs which only access the SQLite DB, URIs which also communicate with the scanner device and finally, URIs with human interactivity:

- **SQLite** (*GET domain/fingerprints*, *GET domain/fingerprints/id*): the response time of the SQLite database is very fast. Response time of the overall embedded system is very good.
- **SQLite + scanner device communication** (*DELETE /fingerprints*, *DELETE /fingerprints/id*): communication time is high. The time between opening the serial port and sending and message handshaking (receiving packet commands) is about 2 s. The serial port *ttyAMA0* of the *Raspberry* can work at 9600 and 115,200 baud speeds. It was set at 115,200 baud. Overall, we have a robust but somewhat slower communication due to the limitations of the serial communication interface.
- **SQlite + Device communication + Human interactivity** (*POST /fingerprints*, *PUT /fingerprints/id*, *GET /fingerprints/identify*): in this category, the times are the longest due to human interaction.
  We compared the Enroll process time of the GT511C1R Fingerprint scanner (7.1 s) with the one obtained in a Oukitel u8 smartphone (10–15 s) with Android and an iPhone 6 (15–20 s). Thus, the GT511C1R Enroll time was the fastest. In the GT511C1R device, the Identify process was 4.5 s and the Ouktiel and iPhone took 2 and 1 s respectively. In this case, the GT511C1R Identify performance was the worst. We can conclude that the GT511C1R times are so good in terms of system usability. Actually, they are very competitive with current smartphone devices.

Significant differences between different categories can be observed. Although the overall assessment was satisfactory, there is room for improvement. In general, we can say that the system client-server-device communication worked properly.

### 5.2. GT511C1R Fingerprint Analysis

Table 3 shows the successful tries of the principal scanner processes, *Identify* and *Enroll*. In both operations, the number of successes in the first try is quite high compared with the remaining attempts. The efficiency of the scanner is in the 70%–80% range. However, the scanner behaved properly as long as the fingerprint occupied almost 100% the scanner area and it was pressed hard enough. The results reached the same conclusion after repeating 10 Identifications and 10 Enrolls.

**Table 3:** Identify and Enroll tries.

Routine	1st Time	2nd Time	3rd Time
**Identify**	14	4	2
**Enroll**	15	5	0

Another test was to identify the finger occupying less than 50% of the scanner area 10 times. The scanner did not pass the test, failing to identify it on any of the 10 tries. When there was a failure, the scanner always sent back an error packet.

Finally, we compared the effectiveness of the scanner GT511C1R with other scanners from a Oukitel u8 and iPhone (touchID) smartphone devices. Table 4 shows the times that both devices processed the Identify and Enroll operations correctly in 10 attempts. The results were similar as long as the scanner was in the same position. Unlike the smartphone scanner, the GT511C1R only operated properly in a horizontal position on a physical support. It needed to be fixed to a physical platform.

**Table 4:** Comparison of the effectiveness of the *gt511c1r*, Oukitel u8 and iPhone 6 scanners.

	No Movement			Movement		
	*gt511c1r*	u8	iPhone	*gt511c1r*	u8	iPhone
**Identify**	7/10	8/10	10/10	2/10	7/10	9/10
**Enroll**	8/10	10/10	10/10	1/10	8/10	10/10

### 5.3. Analysis of the Node.js webServer

**Figure 6:**
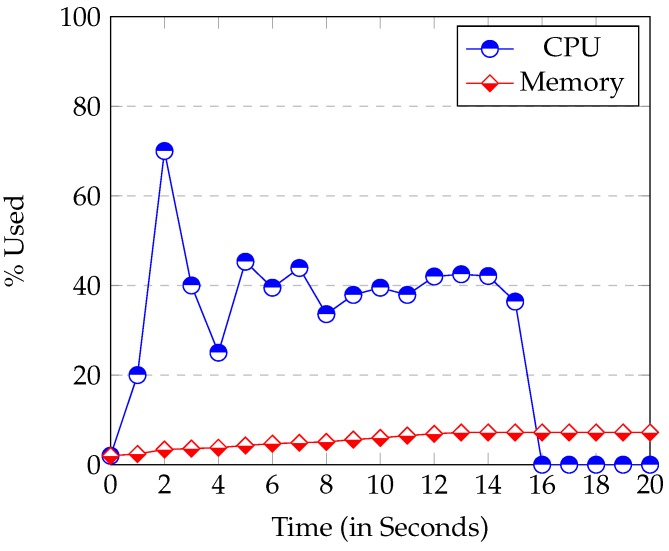
*Raspberry* CPU and Memory Usage at server’s start up.

The node *Node.js* uses considerably more CPU than the *Raspberry* at start up. Its execution usually reached 50% of CPU or more (see Figure 6). This high CPU usage is caused by the loading and start up of modules. All modules are loaded at the beginning of the application. It can be seen in Figure 6 that the start-up time in the *Raspberry* was between 15–20 s. The start up time of the server instead (executed in a laptop), was never longer than 5 s. So, the start-up time is bounded by the device speed.

The Memory used at start-up is proportional to the modules used and the database size. Once the start-up phase has been completed, the occupation of the CPU drops sharply to nearly 0%. In other words, the CPU usage of *Node.js* is minimum when it is waiting for an event.

**Figure 7:**
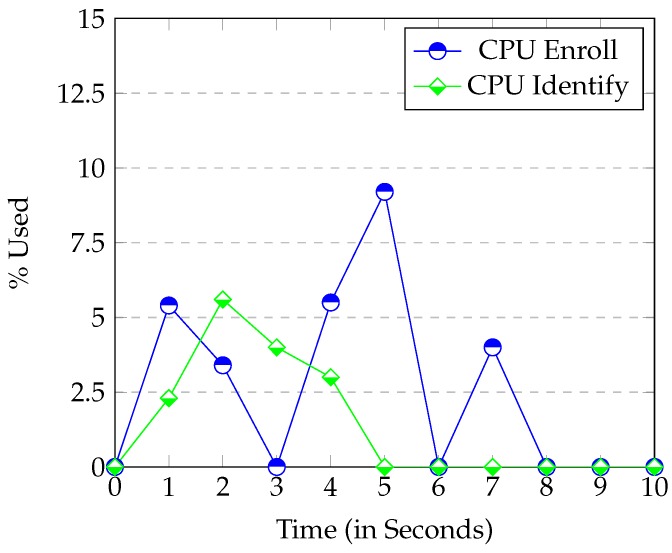
CPU usage when the Enroll and Identify process are called.

**Figure 8:**
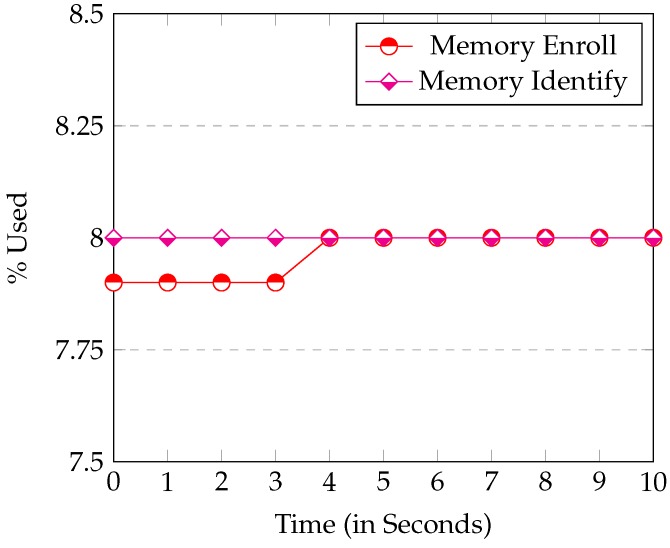
Memory Usage by the Enroll and Identify processes.

Figure 7 and Figure 8 show the CPU and Memory usage respectively in the *Identify* and *Enroll* processes. *Node.js* consumes little Memory or CPU. When Enroll saves a new fingerprint entry in the SQLite database, the Memory used only increases by 0.2%. This result confirms that SQLite database is really small. Thus, it is perfect for systems with limited Memory (like ours). The graph also shows the CPU when the server is communicating with the device. In this case, you can see that the CPU use is always under 12%, so once again, the *Raspberry* executed the web server perfectly.

In conclusion, the behavior of the server performance running on the *Raspberry* embedding device worked well. The *Raspberry* has many hardware limitations (*μ*processor ARMv5 and only 1GB of RAM Memory), but this embedded system behaved perfectly as a web server. The only drawback was its high elapsed time at start up.

### 5.4. Client

In this section the response times of the different URIs are evaluated in different web browsers, running on a PC. The client, executing in a *Epiphany* browser, installed in the *Raspberry* was also tested.

#### 5.4.1. Client Running in the *Raspberry*

In this section, the *Epiphany* browser (default browser in *Raspberry*) is evaluated. This browser has to be executed jointly with the Windows system (in the graphic mode) of the *Raspberry*.

**Figure 9:**
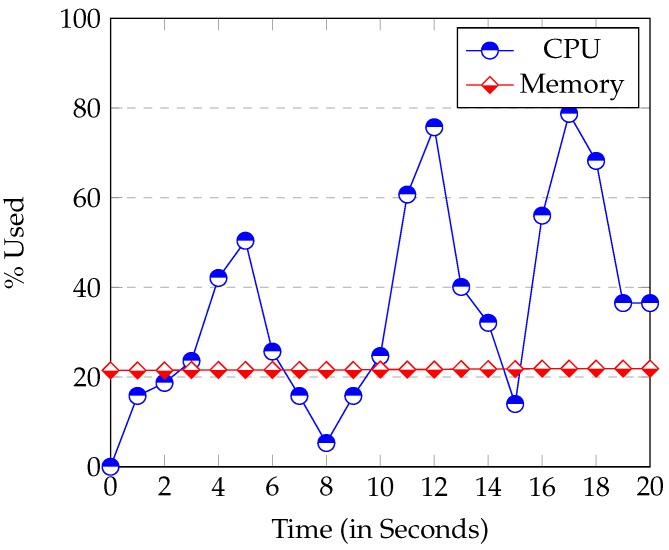
CPU and Memory Usage when the client is executed in the *Raspberry*.

Figure 9 shows the CPU and Memory usage when the client application (*Angular.js*) of the *Epiphany* browser is executed in the *Raspberry*.

It can be observed that the CPU and Memory used increased greatly in Figure 9 compared with Figure 6. When the *Raspberry* browser runs the *Angular.js* client, it consumes a lot of CPU and Memory resources. This can drastically drop both website (client) and device management (server) performance. The *Raspbian* operating system needs to load the X11 Windows system. Thus, it is much better to run the client in another device other than the *Raspberry* with more CPU and Memory. Thus, connecting a screen to the *Raspberry* and executing both the client and the server inside it would not be a good choice.

#### 5.4.2. Choosing a Browser for the Client

This section will evaluates the behavior of a client when running on a PC or laptop because, as we have seen before, executing it in the *Raspberry* (*Epiphany* browser) is not a good option.

We analyzed the execution performance of the *Angular.js* client application in the compatible browsers Google Chrome, Mozilla Firefox, Opera, Epiphany and Midori. The most significant difference between them was the CSS (Cascading Style Sheet) used. However, the CCS used did not affect performance at all.

We measured the response time in sending a request and receiving an answer packet from the server. The request chosen was *GET domain/fingerprints* because it is the one that transmits more information, and thus performance differences can be more easily appreciated. Table 5 shows the mean times obtained in each browser when the request was executed 10 times. Firefox gave the best results. Surprisingly, it was one order of magnitude faster than the remaining 3. This difference can cause significant delays in the overall system. So, gains from choosing Firefox can even overcome the choice of more expensive devices.

**Table 5:** Browser performance.

	Chrome	Firefox	Opera	Midori	Epiphany
Time (in ms)	36	22	32	30	38

### 5.5. Precision and Recall Analysis

In this section, fingerprint recognition was evaluated. In doing so, the Precision, Recall and F scores were used, which are based on the following fingerprint sampling possibilities:

- *True positive (tp):* a user registered in the database puts a finger on the scanner and the system recognizes him/her.
- *False positive (fp):* an unregistered user puts a finger on the scanner and the system recognizes him/she.
- *True negative (tn):* an unregistered user puts a finger on the scanner and the scanner does not recognizes him/her.
- *False negative (fn):* a user registered in the database puts a finger on the scanner but the system does not recognizes him/her.

Precision and Recall are defined by the Equations (1) and (2) respectively.

(1) $$Precision=\frac{tp}{tp+fp}$$

(2) $$Recall=\frac{tp}{tp+fn}$$

Table 6 and Table 7 show the results obtained with 100 trials. Table 6 shows the results obtained with 15 users registered in the database (50 random trials were made), so only true positives and false negatives were sampled. The results obtained were satisfactory. The application answered the 74% of the trials correctly. Table 7 shows the true negatives and false positives obtained also with 50 trials but, in this occasion randomly chosen between 15 unregistered users. As can be seen, the system never failed. It achieved a 100% success rate. Based on these results, the Precision and Recall were 1 and 0.74 respectively. Keeping in mind the low-cost of the system implemented, these results demonstrate its high performance in accordance with the Precision and Recall. These two indices are close to 1. In the case of Precision, that is equal to 1, the best possible result.

**Table 6:** Trials with users already registered in the database.

True Positives	False Negatives	Total Trials
37	13	50

**Table 7:** Trials with users not registered in the database.

True Negatives	False Positives	Total Trials
50	0	50

Once Precision and Recall have been defined, it is important to calculate the F score. F (defined in Eequation (3)) is a metric that gives a harmonic mean of Precision and Recall. F scores also range between [0…1]. Also, the best values are closer to 1.

(3) $$F=2\times \frac{Precision\times Recall}{Precision+Recall}$$

Returning to our example, by applying the Equation (3), an F score of 0.85 was obtained. As 0.85 is very close to 1, it can be said that the obtained F was a good score.

### 5.6. ROC Curve

We also obtained the ROC curve of the system performance with 10 users (see Table 8). Half of them are genuine and the others are impostors. With these users a different number of trials were carried out. Attempts per user were ten times its order on the list. For example, User 1 (User 10) performed 1 × 10 = 10 (10 × 10 = 100) attempts. Users 1, 2, 5, 6 and 10 were genuine users and the remaining ones were impostors. Column Success1 shows the results obtained when the system failed (0) and when it never failed (1). We considered a system fault to be when it gave at least one false positive or when half the trials were false negatives. Column Success2 considered it a system fault only when it gave at least one false positive.

**Table 8:** User attempts and successes.

User	Attempts	Success1	Success2
User 1	10	0	0
User 2	20	0	0
User 3	30	1	1
User 4	40	1	1
User 5	50	1	1
User 6	60	0	1
User 7	70	1	1
User 8	80	1	1
User 9	90	1	1
User 10	100	1	1

**Figure 10:**
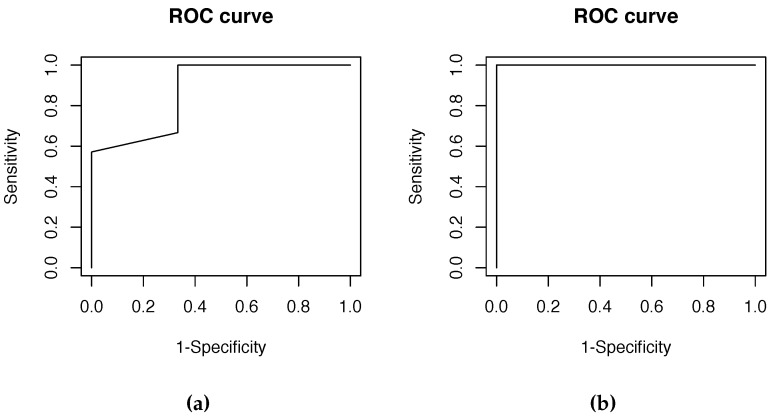
ROC curves. (**a**) ROC curve Success1; (**b**) ROC curve Success2.

Figure 10a,b show the ROC curve for the Success1 and Success2 cases respectively. These results were excellent. In the Success1 case, we evaluated any kind of system error, and so it is obvious that there was more chance of error than in Succcess2. However, the score was very satisfactory (Area Under the Curve, AUC = 0.87). Note that in the Success2 case, we evaluated the effectiveness when the system denied access to unauthorized users. In this occasion, the behavior obtained was perfect (AUC = 1). However, we can say that the scores in both cases were excellent. So we can conclude that the *FingerScanner* system presented works properly.

## 6. Conclusions and Future Work

The conclusions are based on the project results. The results were good and proved that *FingerScanner* could be embedded in a commercial product.

The system architecture is well designed. It allows connections to the *FingerScanner* from different devices. The front-end (client) can be executed in any computer or mobile devices with a browser. Functionality is easily extensible. These features make the *FingerScanner* system robust and flexibile.

The serial communication server device is slow (115,200 baud). However, it is acceptable, as it does not greatly penalize the overall system performance. It must be said that the scanner device was a good choice, because it gives a good efficiency/cost relation, provided that the sensor is in a static position. In addition, the finger recognition success rate of the FingerScanner system obtained an F score of 0.85.

The *Raspberry* hardware in this project is robust, and it works properly. Its viability was carefully tested, because the CPU and Memory of the *Raspberry* have several restrictions in computational power and capacity, respectively. However, the CPU behaved properly for its service requirements. Furthermore, the FingerScanner application fit in the *Raspberry* Memory very well.

The website (client) security should be improved, for example, by using the https protocol and iptablesscripting, so the system will become safer. The transmission of information should also be encrypted. *Node.js* and *Angular.js* have modules like *crypto* to encrypt. Thread concurrency issues must also be solved by using event-driven controllers, for example.
